# Regeneration difficulties in patients with FQAD can limit the use of iPSc-based cell therapy

**DOI:** 10.1186/s13287-022-02886-0

**Published:** 2022-05-21

**Authors:** Dagmara Grot, Katarzyna Wasiak, Jerzy Tyszkowski, Ewelina Stoczynska-Fidelus, Tomasz P. Ochedalski, Piotr Rieske

**Affiliations:** 1 Department of Tumor Biology, Medical University of Lodz, Zeligowskiego 7/9, 90-752 Lodz, Poland; 2 Department of Research and Development, Personather Ltd., Inwestycyjna 7, 95-050 Konstantynow Lodzki, Poland; 3 Fluoroquinolone Toxicity Study NFP, 6444 W. Belmont Ave. Unit B, Chicago, IL 60634 USA; 4 Department of Molecular Biology, Medical University of Lodz, Zeligowskiego 7/9, 90-752 Lodz, Poland; 5 Department of Comparative Endocrinology, Medical University of Lodz, Zeligowskiego 7/9, 90-752 Lodz, Poland

**Keywords:** Fluoroquinolones, FQAD, Induced pluripotent stem cells, Reprogramming

## Abstract

Etiopathogenesis of fluoroquinolone-associated disability (FQAD) syndrome is not fully understood, yet research could progress by utilizing induced pluripotent stem cells (iPSc) from people with this syndrome. Similarly, iPSc, or rather their derivatives, could be used in their therapy, not only for FQAD but also for other disorders in which generated autologous iPSc and their derivatives might be helpful. Urine was collected from ten donors with FQAD, and reprogramming of these cells was conducted with the use of Epi5TM Episomal iPSC Reprogramming Kit. IPSc were generated in one out of ten person’s urine cells. While urinary cells are considered the easiest mature cells to be reprogrammed into iPSc, the urinary cells from six consecutive donors quickly became senescent. Stable urine primary cell cultures could not be obtained from the three remaining donors. Repeated attempts to reprogram epithelial cells were not successful. During parallel studies conducted for healthy donors, reprogramming success was achieved in six out of ten cases. These data may suggest serious limitations in the regeneration system of individuals with FQAD. Consequently, it indicates that therapy with autologous iPSc derivatives may face serious difficulties in their case, still, the first iPSc cell line from a person with FQAD was established.

## Introduction

Quinolone antibiotics kill bacteria by inhibiting enzymes called class II topoisomerases. These enzymes are involved in untangling DNA during cell proliferation. Quinolones bind to these enzymes, thus preventing normal enzyme reactions [1].

In the 1980s, researchers modified quinolones by adding fluorine atoms to the compound structure, increasing these antibiotics penetrance into tissues. Penetrated tissues include the central nervous system and cardiac tissues, which improve effectiveness against bacterial infections. These actions, however, also caused death and damage to organs such as the liver. Therefore, some FDA-approved fluoroquinolones (FQ) were withdrawn from use [2]. Still, many patients suffer after using approved antibiotics, developing enigmatic and quite a severe spectrum of side effects, finally classified by the FDA as fluoroquinolone-associated disability (FQAD) [3]. In the case of FQs, it is suspected that symptoms are caused by mitochondrial [4, 5] and genomic DNA [6–8] damage. To this end, we attempted to develop an induced pluripotent stem cell (iPSc) model to study this disease and verify whether reprogramming technology can be used in the future to treat patients with FQAD and their other disorders in which autologous-induced pluripotent stem cells and their derivatives may be used. Urinary cells are considered as relatively easy to reprogram [9]; unfortunately, iPS cells could not be easily generated from these patients’ somatic cells. This raises additional concern about global FQ use and the accessibility to iPSc-derived treatments for FQAD patients.

## Materials and methods

### Cell culture

Epithelial cells were isolated from urine samples according to the protocol described previously [10]. Briefly, 100 mL of urine sample was collected, transferred into a 50-mL conical tube and centrifuged at 400×*g* for 10 min at room temperature. The supernatant was removed; the cell pellet was washed twice with 25 mL of PBS supplemented with penicillin (100 U/mL), streptomycin (100 µg/mL), amphotericin B (0.25 µg/mL) and centrifuged again. The supernatant was discarded, and the cell pellet was suspended in Renal Epithelial Cell Growth Medium (REGM BulletKit, Lonza) and plated on gelatine-coated cell culture plates (Attachment Factor Protein, Life Technologies). After reaching 90% confluency, cells were passaged with TrypLE Select (Life Technologies) into a new well for further expansion.

Induced pluripotent stem cells were cultured according to Drozd et al. [9].

### Reactive oxygen species detection

A cellular reactive oxygen species (ROS) assay kit (Abcam, ab186027) was used to determine ROS levels, according to manufacturer’s protocol. Statistical analysis was performed using GraphPrism 5 software. Comparisons among groups were performed using One-way ANOVA with Dunnett’s multiple comparison test. Error bars indicate SD (*n* = 3). *P* < 0.05 was considered statistically significant.

### Reprogramming of urinary epithelial cells into iPSc and following differentiation

IPS cells were generated as described previously [9]. Urinary epithelial cells were seeded at a density of 8 × 10^4^ per well of a six-well plate coated with Geltrex basement matrix. Cells were maintained in REGM medium. After overnight incubation, the culture medium was replaced with a fresh one, and cells were transfected with 2 μg of episomal plasmids (Epi5™ Episomal iPSC Reprogramming Kit, Life Technologies), 400 ng of each: pCE-hOct3/4, pCE-hSK pCE-hUL, pCE-mp53DD, pCXB-EBNA1 and 1 μg of an additional plasmid to increase efficiency—pCE-mCherry-miR302/367. FuGENE HD transfection reagent (Promega), at a 3:1 reagent-to-DNA ratio, was diluted in pre-warmed Opti-MEM medium and incubated for 5 min at room temperature. Plasmid DNA was added to the mixture up to a total volume of 100 μL and incubated for 30 min. The solution of the complexes was added in a dropwise manner directly to cells grown in one well of a six-well plate in 2 mL of medium.

The next day, the culture medium was replaced with TeSR-E7 medium (StemCell Technologies), and the transfection was repeated as previously. TeSR-E7 medium was changed every day up to two weeks post-transfection. On day 15, the culture medium was changed to Essential 8. The medium was replaced daily for the next two weeks. Within twenty to thirty days post-transfection, iPSCs expanded to a size appropriate for transfer. Colonies were transferred onto new Geltrex-coated culture dishes and further propagated in Essential 8 medium.

Finally, differentiation of iPS cells into three germ layers was conducted as described previously [9].

### Immunofluorescence analysis

For the immunocytochemical analyses, iPSc or other cells were seeded on Geltrex-coated glass coverslips. Cells were fixed in 4% paraformaldehyde in PBS for 20 min (iPSc) or 15 min (differentiated cells) and permeabilized with 0.25% (iPSc) or 0.1% (differentiated cells) Triton X-100 in PBS for 10 min at room temperature. Next, preparation was performed as already described [9] (Table 1).

**Table 1:** Antibodies used for immunocytochemical staining

Antibody	Host	Manufacturer	Dilution
anti-SOX2	Rabbit	Abcam, ab97959	1:500
anti-OCT3/4	Mouse	Santa Cruz Biotechnology, sc-5279	1:500
anti-TRA-1-60	Mouse	Invitrogen, 41-1000	1:100
anti-TRA-1-81	Mouse	Invitrogen, 41-1100	1:100
anti-αSMA	Mouse	R&D Systems, MAB1420	1:250
anti-MAP2	Rabbit	Abcam, ab32454	1:500
Anti-SOX17	Rabbit	Millipore, 09038	1:100
anti-mouse Alexa Fluor 594	Donkey	Invitrogen, A21203	1:500
anti-rabbit Alexa Fluor 488	Donkey	Invitrogen, A21206	1:500

## Results

### Isolation and propagation of urine-derived epithelial cells

**Fig. 1:**
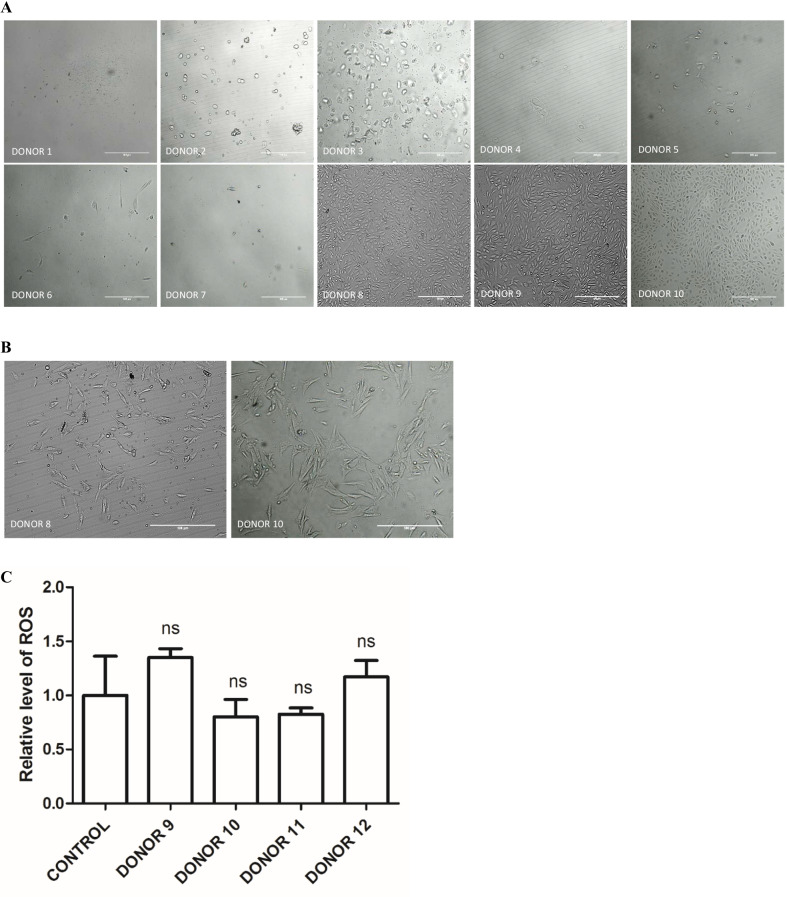
Urinary cells cultures and analyzes. **a** Cells isolated from 10 donors. For donors 1–3, proliferating epithelial cells could not be obtained. In case of donors 4–7 isolated epithelial cells became senescent. For donors 8–10 stable, proliferating urinary cell cultures were obtained. **b** Cells from donor 8 and 10 became senescent during reprogramming. For better readability, the light microscopy images were contrast and brightness enhanced. Scale bars represent 500 μm. **c** Detection of the ROS levels in urine samples (*n* = 3), *ns* not significant

Urine samples were collected from ten donors with FQAD (Fig. 1a, Table 2). All tested individuals had good health before FQ prescription therapy. For three donors (DONOR 1–3), stable urine primary cell cultures could not be obtained. The isolates from another four out of ten donors (DONOR 4–7) contained single viable epithelial cells which became senescent very quickly. Two of the stable primary cell cultures (DONOR 8 and DONOR 10) became senescent right after transfection with reprogramming episomes (Fig. 1b). Finally, urinary epithelial cell cultures derived from three out of ten individuals with FQAD were suitable for being subjected to the process of reprogramming. Only one donor provided cells that were successfully reprogrammed. During parallel studies conducted with healthy donors, success was achieved in six out of ten cases [9].

**Table 2:** Information on donors—course of FQ treatment, symptoms of FQAD, effect of the experiment

Record	Age range	FQ name/days of treatment/dose in mg per day	Time since last FQ treatment	ADR after FQ immediate/delayed	FQAD symptoms constant/intermittent	Test result
DONOR 1	30–39	Lev/9 days/500 mg	1 year	Immediate	Constant	Failed/no adherence
DONOR 2	70–79	Lev/2 days/500 mg	10 years	Immediate	Constant	Failed/no adherence
DONOR 3	50–59	Lev/7 days/500 mg	6 years	Delayed	Constant	Failed/no adherence
DONOR 4	30–39	Cip/10 days/500 mg	1 year	Delayed	Constant	Failed/senescence before reprogramming
DONOR 5	20–29	Cip/30 days/1000 mg	0.5 year	Delayed	Constant	Failed/senescence before reprogramming
DONOR 6	30–39	Cip otic/10 days/3 mg	0.5 year	Delayed	Constant	Failed/senescence before reprogramming
DONOR 7	40–49	Cip otic/10 days/6 mg	0.5 year	Delayed	Constant	Failed/senescence before reprogramming
DONOR 8	60–69	Cip/10 days/ 1000 mg	10 years	Delayed	Constant	Failed/senescence after reprogramming
DONOR 9	50–59	Lev /5 days/ 500 mg	9 years	Delayed	Constant	Complete
DONOR 10	40–49	Lev/24 days/500 mg	5 years	Delayed	Constant	Failed/senescence after reprogramming

*Cip* Ciprofloxacin, *Lev* Levofloxacin

### Analysis of oxidative stress level

Due to the widespread senescence of cells during cultivation or reprogramming, an analysis of oxidative stress level was performed. Comparison of selected samples from individuals with FQAD (donor 9, donor 10, and additional samples from donor 11 and donor 12, for which the reprogramming attempt was not been taken yet) to the control group (healthy donor) showed no statistically significant differences in the level of oxidative stress (Fig. 1c).

### Reprogramming of urine-derived epithelial cells

**Fig. 2:**
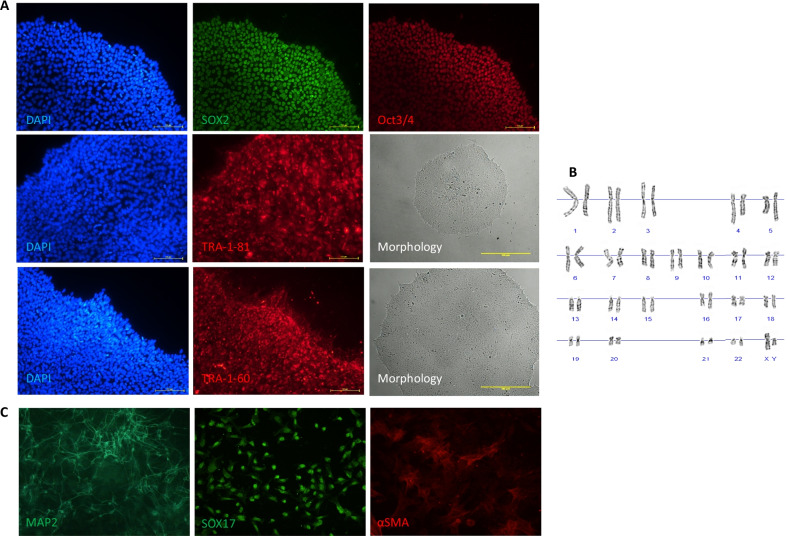
Characterization of the iPS cells from donor 9.** a** Representative images showing iPSc generated from donor 9 urinary cells immunostained with pluripotency markers SOX2 (green) and OCT3/4 (red), TRA-1-60 (red), TRA-1-81 (red) and morphology of the cell culture under self-renewal conditions. **b** Karyotype analysis indicated karyotypically normal iPS cells. **c** Representative images showing iPSc differentiation into three germ layers: MAP2 (green)—ectoderm, SOX17 (green)—endoderm, αSMA (red)—mesoderm. Each image was taken at magnification 200×. For better readability, the light microscopy images were contrast-and brightness-enhanced. Scale bars represent 100 μm and 500 μm (immunofluorescence and bright field images, respectively)

Cell reprogramming was performed using the forced expression of the transcription factors NANOG, OCT3/4, KLF4, SOX2, L-MYC and LIN28, introduced using a non-viral episomal system based on EBNA-1/oriP elements. In order to increase the efficiency of the reprogramming process, vectors encoding the 302/367 microRNA and mutated p53 protein were also included. All three successfully stabilized urinary cell lines were subjected to transfection (DONOR 8–10). IPSc were generated only from one donor (DONOR 9), and, in the other two (DONOR 8 and DONOR 10) cases, cells became quickly senescent (Fig. 1b). In the latter two cases, repeated attempts to reprogram were not successful. IPSc colonies started to form on day 16 after the initial transfection and were picked up mechanically from the plate on day 21. Colonies were further propagated until passage 10. Stable iPS cells were analyzed for the presence of pluripotency-associated markers. To verify iPSc identity, the immunofluorescence assay was conducted by using anti-OCT3/4-, anti-SOX2-, anti-TRA-1-60- and anti-TRA-1-81-specific antibodies (Fig. 2a). Obtained iPSc maintained a normal human karyotype (Fig. 2b) (karyotype analysis performed by GENOS Company, Poland).

### Differentiation of generated iPSc into germ layers

A stable culture of obtained iPSc was routinely differentiated into germ layers. Immunofluorescence analysis showed the expression of markers characteristic for the endoderm—SOX17, mesoderm—αSMA and ectoderm—MAP2 (Fig. 2c).

## Discussion

The effect of fluoroquinolones on cells and tissues is poorly understood. IPS cells have become a valuable research model for many diseases. In the case of FQs, it is suspected that symptoms are caused by mitochondrial and genomic DNA damage [4–8]. The influence of these changes on cells can be tested, with the use of an iPS cells model. IPS cells may also become a potential therapeutic tool for patients with FQAD. Starting from iPSc, regenerative therapy could be carried out, beginning with the typical tendon damages found with FQAD. In the case of mitochondrial damage, it is worth considering the selection of iPS cells with the highest percentage of the normal mitochondria for therapeutic purposes. Such an approach could possibly allow for the selection of suitable cells to develop advanced therapeutic medicinal products.

Urine cells are well known to be very accessible and easy to reprogram. Drozd et al. [9] showed that these cells generate a higher number of iPSc colonies in comparison with skin cells (urine cells are 100 times more efficient). Scar cells were the most difficult to reprogram (about 500 times less efficient than urine cells), which could have been an issue to use in this study as FQAD patients frequently show structural skin damage. According to Drozd, et al., epithelial phenotypes of urine cells are most likely pro-reprogramming. It is well known that fibroblastic, but not epithelial cells, must go through MET during reprogramming. Finally, the subpopulation of urine cells shows TRA-1-60 and TRA-1-81 expression [9]. The reprogramming efficiency of blood cells is similar to fibroblast reprogramming efficiency [11]. All the above suggests that other cells can be more difficult to reprogram than urine cells when it comes to cases of FQAD; however, we cannot exclude some unique damage to the kidney in this syndrome. ROS analysis showed no differences between cells from healthy donors and cells from FQAD patients, suggesting that oxidative stress, in this case, is not directly related to cell senescence and failure of reprogramming.

This study showed that it is very difficult to generate iPS cells from urine epithelial cells of patients with FQAD. Equally important is the fact that the efficacy of the cell culture establishment was very low, with only one out of ten patients providing cells suitable for reprogramming. This is surprising as other previous studies showed that urine cells should be a very efficient source for reprogramming [9, 12]. An important fact about FQAD patients is that their connective tissue is damaged; however, this study also suggests that renal structures can be preferentially damaged by FQ’s. It has to be emphasized that so far, no one has been able to define which exact type of cells from urine become reprogrammed; however, in our previous research we detected cells showing markers for stem cells [9]. Verification of the presence and the percentage of these cells in urine from FQAD individuals should be considered. If that premise is accurate, it would serve as a marker for the malfunctioning of regenerative systems.

It seems that there is no easy model of cell reprogramming when studying this syndrome, which might limit research opportunities. Future efforts to apply regenerative medicine to FQAD individuals based on reprogramming technology will be a challenging process that will need to be refined. The fact that this study was able to establish the first model of an iPS cell line from a person with FQAD may provide hope for the creation of future treatments.

## Data Availability

All data generated or analyzed during this study are included in this published article.

## Abbreviations

DMEM: Dulbecco’s modified eagle medium
EDTA: Ethylenediaminetetraacetic acid
FBS: Fetal bovine serum
FDA: Food and Drug Administration
FQ: Fluoroquinolone
FQAD: Fluoroquinolone-associated disability
iPSc: Induced pluripotent stem cells
MEM: Minimal essential medium
PBS: Phosphate-buffered saline
REGM: Renal cell growth medium
ROS: Reactive oxygen species
RPMI: Roswell Park Memorial Institute Medium
